# Editorial: Interactions between natural and built environment microbiomes in a One Health context

**DOI:** 10.3389/frmbi.2025.1608228

**Published:** 2025-05-07

**Authors:** Weitao Shuai, Bridget Hegarty, Alexander Mahnert, Erica Marie Hartmann

**Affiliations:** ^1^ Department of Civil & Environmental Engineering, Northwestern University, Evanston, IL, United States; ^2^ Center for Synthetic Biology, Northwestern University, Evanston, IL, United States; ^3^ Civil and Environmental Engineering, Case Western Reserve University, Cleveland, OH, United States; ^4^ Diagnostic & Research Institute of Hygiene, Microbiology and Environmental Medicine, Medical University of Graz, Graz, Austria; ^5^ Department of Medicine, Division of Pulmonary Medicine, Northwestern University, Evanston, IL, United States

**Keywords:** built environment, One Health, microbiome, antimicrobial resistance, quantitative microbial risk assessment (QMRA)

Humans, particularly in industrialized societies, spend an estimated 90% of their time within built environments, such as homes, workplaces, and urban infrastructure (Klepeis et al., 2001). These spaces play a critical role in shaping health outcomes and warrant deeper scientific exploration. Built environments accumulate microbial and chemical components from adjacent natural environments, particularly through water systems, as well as air exchange and human and/or animal activity (Rai et al., 2021). The built environment then shapes microbiomes via anthropogenic pressures such as disinfection practices and material selection. These microbiomes then serve as a source of exposure for human occupants and can also reenter natural environments, creating feedback loops that alter both environments. Sessitsch et al. depict this interaction as a network, showing microbiome transfer between environments including the built environment (Sessitsch et al., 2023). This Research Topic adopts a One Health perspective to examine these bidirectional interactions, emphasizing their implications for human, animal, and ecosystem health.

In recent years, the importance of the One Health framework has been recognized globally. Studies have addressed the role of the built environment microbiome in human and animal health (Bosch et al., 2024). However, our understanding of the mechanisms and processes governing natural and built environment microbiome interactions remains minimal, hindering our power to combat critical health threats including antimicrobial resistance (AMR) and emerging pathogens.

Our Research Topic provides insights into important natural and anthropogenic factors shaping the built environment microbiome and their implications in health improvement through four original research articles and one systematic review. Collectively, they emphasize that the “built environment” should not be viewed as a monolithic entity. The collected original research reveals how microbiome composition and health risks vary across functionally distinct spaces – from premise plumbing systems to bathroom fixtures to construction materials – driven by differences in moisture, material chemistry, and occupant behavior.

Scaturro et al. investigate bacterial communities in premise plumbing systems and their associations with *Legionella*. As a wide-spread problem with measurable impacts on human health, better understanding of *Legionella* in premise plumbing systems is needed to control proliferation. The authors identify consistent positive and negative correlations between specific bacterial taxa and culturable *Legionella*. These patterns persist even in systems with varying physicochemical conditions, suggesting that ecological relationships in a microbial community play a critical role in *Legionella* proliferation, which provides potential targets for developing sustainable control strategies.

Huttelmaier et al. explore phage-bacteria interactions in showerhead and toothbrush biofilms, uncovering distinct viral communities shaped by their bacterial hosts. They focus on the less documented and sparsely investigated viral component within the built environment microbial communities. The authors detect novel, uncharacterized phages in these niches, with phage diversity linked to localized bacterial community structure. They propose that environmental factors (e.g., water chemistry, usage frequency) indirectly modulate phage populations by altering host bacteria community. By highlighting how viral communities are even more variable than bacterial communities in built environment microbiomes, these findings emphasize the need to map viral dark matter in One Health frameworks.

Pitell and Haig evaluate the efficacy of antimicrobial showerheads in mitigating drinking water-associated pathogens that can cause infections in the immunocompromised (DWPIs). They focus on real-world conditions and the poorly understood steps in the DWPI transmission pathway: aerosolization and mitigation approaches. Two types of antimicrobial showerhead (antimicrobial filter-based and silver-embedded) are compared with conventional plastic showerheads, but no significant differences are observed in the resulting water chemistry and DWPI presence or abundance. Rather, the study reveals that aerosolization of DWPIs is species-specific, and that showerhead age is the most influential factor to explain DWPI abundance. The results underscore the need to rethink “antimicrobial” material claims and temporal characterization of DWPI aerosolization.

Mhuireach et al. investigate the interplay between microbial colonization and volatile organic compound (VOC) emissions from coated and uncoated cross-laminated timber (CLT) blocks under varying wetting durations. VOC levels spiked immediately after microbial inoculation, followed by a decline, but plateaued during wetting periods. Uncoated CLT mainly emitted terpenes, while coated CLT additionally emitted VOCs associated with coatings, plastics, and industrial solvents. Notably, uncoated CLT had higher microbial abundance compared to coated CLT, possibly due to biocidal substances in coatings. These findings reveal a critical trade-off: although coatings may reduce moisture-driven microbial growth, they alter released VOC profiles, underscoring the need to balance material durability with indoor air quality in microbiome-informed building design.

The systematic review by Quon et al. tackles the broad challenge of quantifying AMR gene conjugation rates across environmental, clinical, and agricultural reservoirs. Synthesizing 113 studies, the review reveals that conjugation frequencies span 12 orders of magnitude, with wastewater systems exhibiting the highest transfer rates. These findings underscore anthropogenic activities as key drivers of AMR spread and align with the premise plumbing and showerhead studies in this Research Topic. Critically, the review emphasizes persistent gaps: undercharacterized conjugation dynamics and inconsistently reported data on donor/recipient densities and important factors, both of which limit predictive modeling accuracy. This work calls for standardized framework to improve experimental data reporting, emphasizing the importance of mechanistic studies in quantitative risk assessments at the natural-built environment interfaces.

The studies in our Research Topic collectively advance our understanding of how anthropogenic activities reshape microbiomes and influence health risks at the natural-built environment interface. Yet, as the collected articles underscore, temporal sampling and characterizations of microbiome dynamics are complex and require further investigation. AMR and pathogen control methods demand further development and rigorous efficacy assessment, some potential examples of which are proposed in this Research Topic. This Research Topic both highlights critical knowledge gaps in understanding natural-built environment microbiome interactions and paves the way for exploring practical solutions through a One Health lens.
